# Physiological and biomechanical aspects of the first female finisher in the longest triathlon in the world – Triple Deca in Ultra Triathlon Italy 2024

**DOI:** 10.17179/excli2025-8612

**Published:** 2025-10-29

**Authors:** Sasa Duric, Luciano Bernardes Leite, Pedro Forte, Marilia Santos Andrade, Ivan Cuk, Pantelis T. Nikolaidis, Katja Weiss, Thomas Rosemann, Beat Knechtle

**Affiliations:** 1 Liberal Arts Department, American University of the Middle East, Egaila, Kuwait; 2 Department of Physical Education, Federal University of Viçosa, Brazil; 3 Department of Sports, Higher Institute of Educational Sciences of the Douro, Penafiel, Portugal; 4 Department of Sports Sciences, Instituto Politécnico de Bragança, Bragança, Portugal; 5 Research Center for Active Living and Wellbeing (Livewell), Instituto Politécnico de Bragança, Bragança, Portugal; 6 Department of Physiology, Federal University of Sao Paulo, Brazil; 7 Faculty of Sport and Physical Education, University of Belgrade, Belgrade, Serbia; 8 InterSynergy Research Center, Belgrade, Serbia; 9 School of Health and Caring Sciences, University of West Attica, Athens, Greece; 10 Institute of Primary Care, University of Zurich, Zurich, Switzerland; 11 Medbase St. Gallen Am Vadianplatz, St. Gallen, Switzerland

**Keywords:** swimming, cycling, running, ultra-endurance, pace

## Abstract

Pacing in multi-day long-distance triathlons has been investigated mainly in male athletes. We analyze physiological aspects such as energy expenditure and heart rate changes as well as biomechanical aspects in swimming (*e.g.* strokes per lane) and running (*e.g.* stride frequency, stride length, vertical ratio, vertical movement, ground contact time) in the first and only female triathlete to finish 30 IRONMAN^®^-distance triathlons in 30 days. The split times, lap times for swimming, cycling and running and variables were recorded with Fenix 7 Sapphire Solar with Normalized Power^®^ (NP^®^), Intensity Factor^®^ (IF^®^) and Training Stress Score^®^ (TSS^®^), and were analyzed. The models' estimations for pacing were assessed with R2. Variance (ANOVA) and associative (Pearson and Spearmen) analysis were conducted at a level of significance of 5 %. Swimming pace remained stable throughout the race (linear p = 0.473), cycling pace demonstrated a significant slowdown (third-order polynomial p < 0.001), and running pace significantly improved (third-order polynomial p < 0.001). Energy expenditure slightly decreased in swimming (p = 0.099) and progressively increased for both cycling (p = 0.034) and running (p = 0.044). Moderate-intensity swimming time initially increased and later decreased, with an opposite trend for high-intensity swimming time. Cycling times at both moderate and high intensities slightly decreased. Running showed decreasing moderate-intensity time and increasing high-intensity time, consistent with improved pace. Transition times increased over the race period, with T1 increasing more prominently. Biomechanical parameters in swimming, including total stroke count and SWOLF index, showed increasing trends. Overall, significant differences were observed in running time at moderate intensity (p < 0.001, η^2^ = 0.513), high intensity (p < 0.001, η^2^ = 0.518) and average pace (p < 0.001, η^2^ = 0.603). The athlete spent significantly more time at moderate intensity (p = 0.019 and p = 0.002) and significantly less time at high intensity (p = 0.011 and p = 0.005) running in the initial phase, compared to the middle and final stages of the race. All biomechanical variables decreased slightly in the opening phase of the race but then increased in the middle and final stages of the race. Overall, the results highlight that running was the discipline most affected by physiological and pacing adaptations throughout the race; while cycling and swimming parameters demonstrated weaker or no consistent associations.

## Introduction

Ultra-endurance races such as ultra-marathons (Scheer, 2019[23]) and ultra-triathlons (Sousa et al., 2020[24]) are of increasing popularity. Studies investigating athletes competing in ultra-endurance mainly investigated the trends in participation and performance (Sousa et al., 2020[24]), as well as physiological (Knechtle et al., 2024[11]) and nutritional (Knechtle et al., 2008[12]) aspects or the influence of environmental conditions. Other studies also assessed the psychological effects in athletes' performance (Meckfessel and Ross-Stewart, 2022[19]; Thuany et al., 2023[26]; Colangelo et al., 2023[6]).

Ultra-endurance races, such as ultra-marathons, are known to induce significant fatigue accumulation over time (Jeker et al., 2020[9]; Vernillo et al., 2016[27]; Berger et al., 2024[3]) and a decrease in performance over time (Berger et al., 2024[3]). Fatigue can produce atypical running biomechanics (Clermont et al., 2019[5]). This leads to slower speeds in running in ultra-runners (Berger et al., 2024[3]) as well as in swimming, cycling and running in ultra-triathletes (Knechtle et al., 2024[11]).

Despite growing interest in ultra-endurance sports, there is still a paucity of data on fatigue dynamics and pacing deterioration among triathletes participating in multi-day events extending beyond 10 days (Knechtle et al., 2024[11]). Ultramarathons are relatively new running events, as such, less is known about physiological and biomechanical parameters that underlie ultra-marathon performance (Thompson, 2017[25]). We have, however, little knowledge about running kinematics and running energetics (Howe et al., 2021[8]) in ultra-marathon runners, but not in ultra-endurance triathletes competing in multi-stage races. Running in marathon and ultra-marathon races differs from running a marathon or an ultra-marathon in a triathlon race since triathletes start the run with a certain exhaustion after the swimming and the cycling part (Bentley et al., 2008[1]). Also, it might be different to observe ultra-triathlons when the athlete has to finish all of the swimming (*e.g.,* 10 distances, 38 km) before going to the 10 cycling distances (1800 km) (Lepers et al., 2011[17]), in comparison when to finish the entire IRONMAN^®^-distance triathlon before to go to the next one the next day (Knechtle et al., 2008[13]). In addition, it has been observed in the World Championships Grand Final triathlon that in-race changes in running speed were due to step length rather than step frequency variation (Le Meur et al., 2013[16]).

Regarding the abovementioned gap, for this case study with the first and only female athlete to finish 30 IRONMAN^®^-distance triathlons in 30 days, we analyzed physiological aspects such as energy expenditure and heart rate changes as well as biomechanical aspects in swimming (*e.g.* strokes per lane) and running (*e.g.* stride frequency, stride length, vertical ratio, vertical movement, ground contact time). The results of such analyses will give us insights into how an athlete can endure such a performance with recommendations for future athletes intending to try competing in such an event. We hypothesized that the athlete would slow down during the race including slower movements in both swimming and running.

## Method

### Ethical approval

The study was conducted in accordance with the recognized ethical standards according to the Declaration of Helsinki adopted in 1964 and revised in 2013. This study was approved by the Institutional Review Board of Kanton St. Gallen, Switzerland (EKSG 01/06/2010). The athlete gave her informed written consent for using all her personal data.

### Athlete with experience and pre-race preparation

The athlete is a female ultra-endurance athlete (born 1971, 164 cm, 67 kg) from Austria. In ultra-cycling, she finished Race Across AMerica (RAAM) in 2017 in 12:04:35 d:h:min in second place in the women's race and in 2019 in 12:05:41 d:h:min in third place (www.raamrace.org/). She further set in 2019 a world record in Race Across Australia (3966 km) in 9:12:33 d:h:min and two world records with 13,333 km cycling in 30 days and 3953,42 km cycling in one week. In ultra-triathlon, she set a world record in 2016 in Double Deca one per day in 279:37:48 h:min:s, in 2018 in Quintuple one per day in 84:44:29 h:min:s, in 2022 in Deca one per day in 137:23:43 h:min:s, and in 2023 in Double Deca continuous race in 554:56:32 h:min:s (www.iutasport.com). 

In the race-specific preparation (start beginning 2024), she invested ~ 21 h in training, mainly in cycling. During winter, she invested ~ 15 hours, and during summer ~ 25-30 hours. During winter, she completes mainly indoor cycling on a Tacx^®^ NEO Bike Smart-Trainer (www.garmin.com/de-DE/p/690885). She was swimming on average ~ 7.9 km per week and ~ 34.3 km per month. For cycling, she invested ~ 16 hours per week and ~ 70 hours per month. For running, her weekly running kilometers are at ~ 45.1 km, and her monthly kilometers are at ~ 195.5 km. Monthly cycling kilometers varied between 1069 km (August) and 2073 (June) while monthly running kilometers varied between 81 km (April) and 217 km (June). The training was mainly split into a unit early in the morning before work and a second unit in the evening after work.

### Race

The race was held near Desenzano del Garda in Northern Italy. The athletes had to complete daily, an IRONMAN^®^-distance triathlon covering 3.8 km swimming, 180 km cycling and 42.195 km running. Race headquarters was at the “Le Ninfee” aquatic park, which features a 25 m lane pool (non-heated) and a 1.06 km flat running track on dirt, grass, sand, and asphalt. The 7 km bike loop is at a separate location 11.5 km from “Le Ninfee” by bike, and one has to do this out-and-back and 23 seven-kilometer loops to get in the required 180 km bike ride. The athletes had on average ~ 1000 m of ascents and 1000 m of descents within the 180 km. In running, the athletes had to complete 40 laps. For swimming, she wore a Sailfish wetsuit, for cycling, she used a Ridley Noah SL with Pancho wheels with rims of 8 cm. For cycling, she wore cycling pants from Cocoon and a cushion from Tempur for her bicycle saddle. In the run, she started with HOKA Speed Goat on day 1, but changed then for the remaining 29 days to TOPO ATMOS. At the start, the environmental conditions were quite good with a water temperature of ~22 °C and sunshine. Over the days, the water temperature continuously fell to ~19 °C during the race and ~17-18 °C for the last week. Air temperature varied between 20 and 25 °C depending upon rainy or sunny days. A total of 7 athletes (6 men, 1 woman) started the race on September 5, 2024. The first man dropped out on day 1, the second man on day 3, the third man on day 4, the fourth man on day 5, the fifth man on day 10, and the last man on day 16. From day 17 to day 30, the athlete was alone in the race.

### Data set and data preparation

The race data with split and lap times for swimming, cycling, and running were obtained from the official race website of the race 'Ultra Triathlon Italy' (www.ultratriathlonitaly.com/). For swimming, lap times were recorded manually and the final swim time was provided. Lap times in cycling and running were recorded electronically using a chip system (www.raceresult.com/). The athlete monitored each split discipline (*i.e.* swimming, cycling, and running) of each day using Fenix 7 Sapphire Solar with Normalized Power^®^ (NP^®^), Intensity Factor^®^ (IF^®^) and Training Stress Score^®^ (TSS^®^). The watch was programmed following the instructions of the manufacturer. In swimming, the following variables were recorded: energy expenditure, average pace, best pace, average heart rate, maximum heart rate, total number of strokes, average stroke rate per lane, average SWOLF (SWOLF is calculated from the time plus the number of strokes a swimmer needs for a lane), swim time at moderate intensity and swim time at high intensity. For cycling, energy expenditure, average heart rate, maximum heart rate, average cycling speed, maximum cycling speed, cycling time at moderate intensity, and cycling time at high intensity were recorded. For running, the following variables were recorded: Pace, energy expenditure, average heart rate, maximum heart rate, average performance, maximum performance, average stride frequency, maximum stride frequency, average stride length, average vertical ratio, average vertical movement, average ground contact time, running time at moderate intensity, running time at high intensity, time running and time walking

### Statistical analysis

All data were tested for normality using the Kolmogorov-Smirnov test. Variables that did not meet the normality assumption were analysed using non-parametric statistical methods.

To examine trends in pacing performance and energy expenditure across swimming, cycling, and running throughout the 30-day race, linear, second-order polynomial, and third-order polynomial regression models were applied. The selection of the best-fit model was based on statistical significance (p< 0.05) and coefficient of determination (R²); where, after preliminary analysis the third-order models provided the best fit for nonlinear adaptations. For each discipline, one-way analysis of variance (ANOVA) was used to determine differences between three predefined race phases: the opening phase (days 1-10), the middle phase (days 11-20), and the final phase (days 21-30). These phases corresponds with "Deca" i.e., 10 IRONMAN^®^ distance triathlons, often reported in the triathlon studies and practice (Knechtle et al., 2024[11]). For post-hoc comparisons, Tukey's HSD test was used. In cases where the assumption of homogeneity of variances was violated (as indicated by Levene's test), the Games-Howell post-hoc test was used. Effect sizes were calculated using eta-squared (η²), with values of 0.01, 0.06, and 0.14 interpreted as small, medium, and large effects, respectively. Correlation analyses were performed to assess relationships between average pace, energy expenditure, heart rate, and time spent at moderate and high intensities in each discipline, using Pearson's correlation coefficient (r) for parametric data and Spearman's rank-order correlation coefficient (ρ) for non-parametric data. Correlations were examined both across the entire race and within the three race phases, providing insights into temporal variations in physiological responses. Additionally, biomechanical parameters in running (stride frequency, stride length, vertical oscillation, and ground contact time) and swimming (stroke count, SWOLF index) were analysed for trends using polynomial regression models. Cross-disciplinary correlations between physiological, pacing, and biomechanical variables in swimming, cycling, and running were assessed using Pearson's and Spearman's correlation analyses to identify carry-over effects between disciplines. Finally, lagged correlation analyses were conducted to explore predictive relationships between pacing and energy expenditure on one day and running performance on the following day, further elucidating fatigue carryover and adaptive pacing strategies. All statistical analyses were performed using SPSS version 26 (IBM, Armonk, NY, USA), with statistical significance set at p < 0.05.

## Results

**Figure 1:**
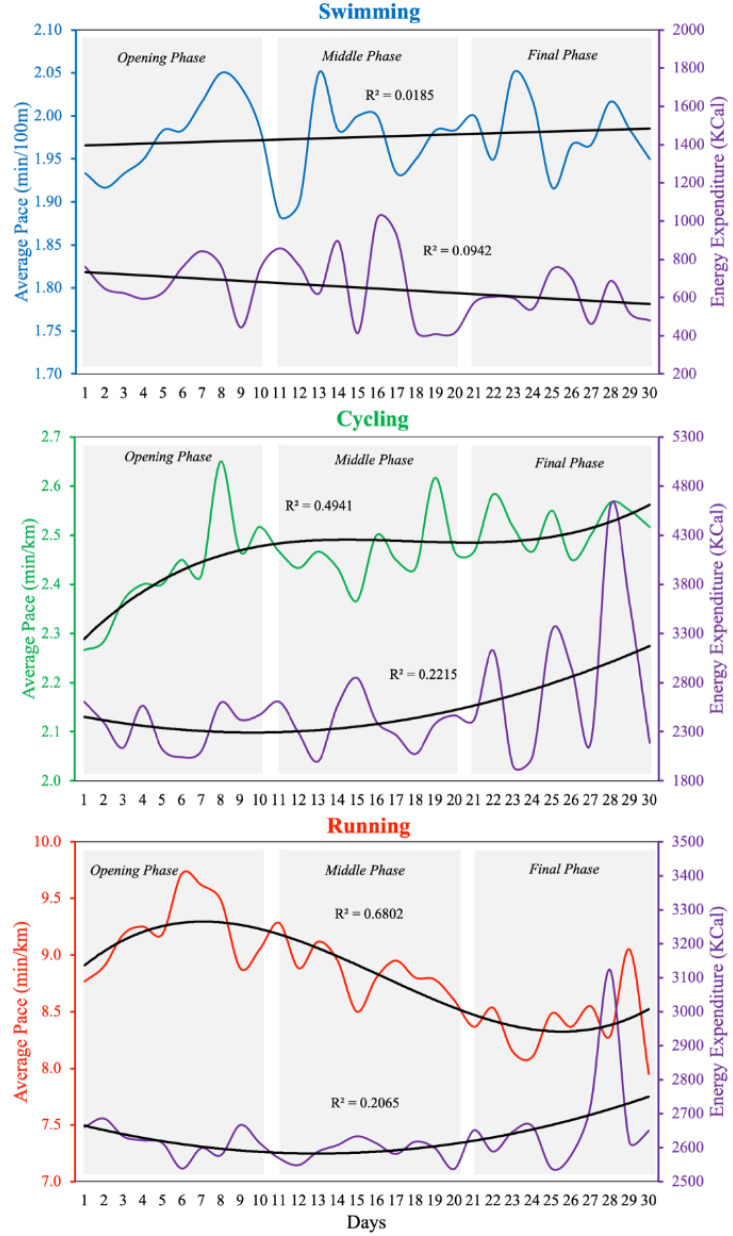
Trends in average pace and energy expenditure during swimming, cycling and running throughout the 30-day Triple Deca Ultra Triathlon.

The average pace and energy expenditure trends, with best-fit linear and polynomial regression trend lines for swimming, cycling, and running, are presented in Figure 1(Fig. 1).

The average swimming pace remained relatively stable across the 30 days (p = 0.473), with no significant linear trend. In contrast, the cycling pace showed a significant deceleration pattern over time (third-order polynomial model, p < 0.001), while the running pace improved significantly as the event progressed (p < 0.001), suggesting a progressive adaptation. Energy expenditure slightly decreased in swimming (p = 0.099) and progressively increased for both cycling (p = 0.034) and running (p = 0.044). According to linear regression models, swimming pace was stable (+0.001 min/100m/day, p = 0.437), cycling pace slowed (+0.006 min/km/day, p < 0.001), and running pace improved (-0.03666 min/km/day, p < 0.001). The average swimming pace remained relatively stable across the 30 days (p = 0.473), with no significant linear trend. In contrast, the cycling pace showed a significant deceleration pattern over time (third-order polynomial model, p < 0.001), while the running pace improved significantly as the event progressed (p < 0.001), suggesting a progressive adaptation.

**Figure 2:**
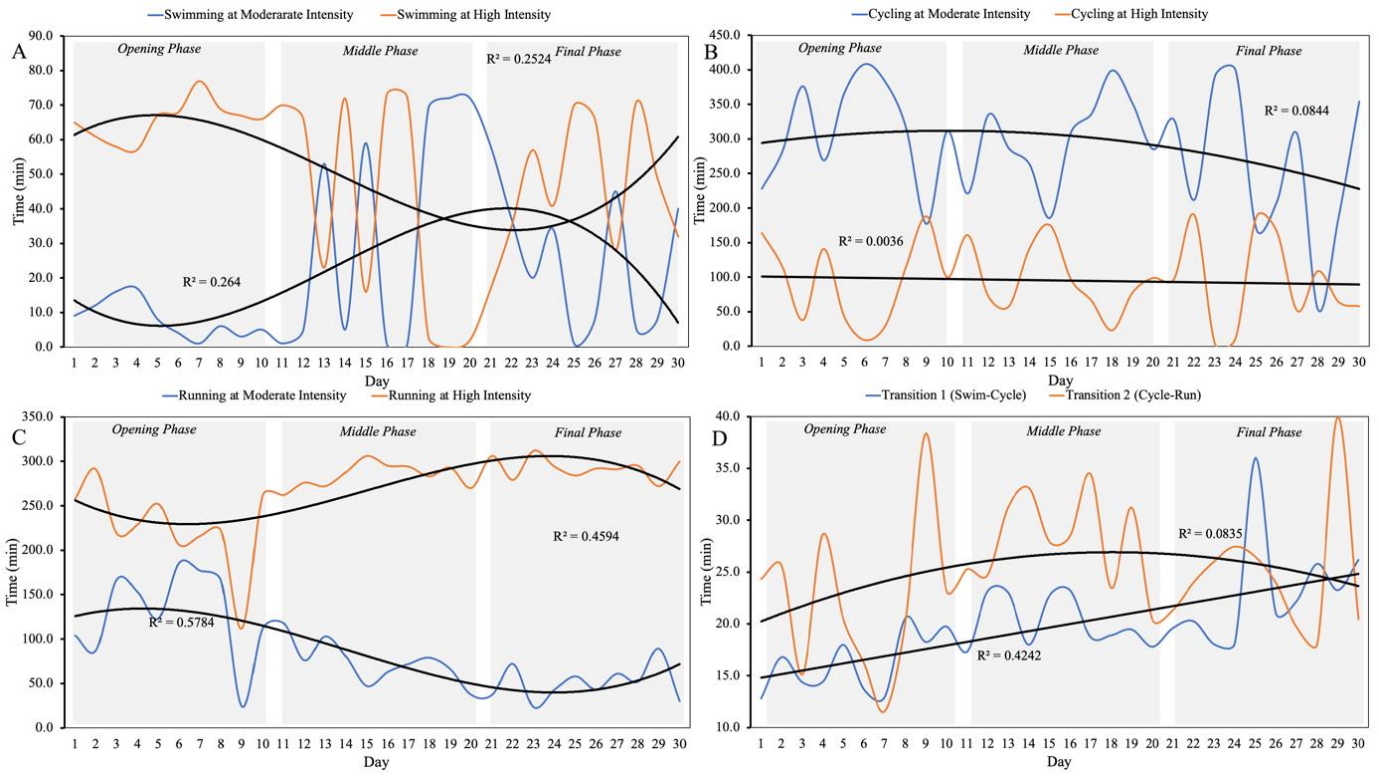
Trends in time at moderate and high intensity in swimming (*panel A)*, cycling (*panel B*) and running (*panel C*), and transition times (*panel D*) over the 30-day race.

Trends in time spent at moderate and high intensities in swimming, cycling, and running, as well as transition times (T1 and T2), are shown in Figure 2(Fig. 2).

Moderate-intensity swimming time initially increased and later decreased, with an opposite trend for high-intensity swimming time. Cycling times at both moderate and high intensities slightly decreased. Running showed decreasing moderate-intensity time and increasing high-intensity time, consistent with improved pace. Transition times increased over the race period, with T1 increasing more prominently.

**Figure 3:**
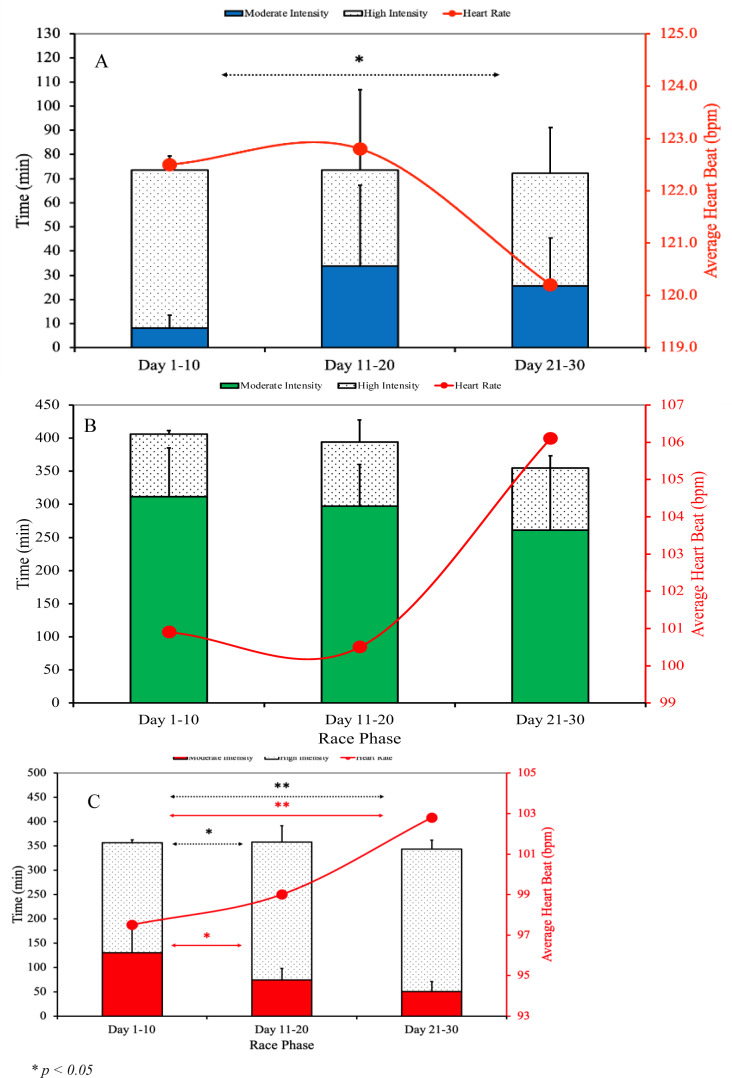
Differences between race phases in average heart rate, moderate and high-intensity time in swimming (A), cycling (B) and running (C) splits.

Differences across race phases in swimming (panel A), cycling (panel B) and running (panel C) are presented in Figure 3(Fig. 3).

**Figure 4:**
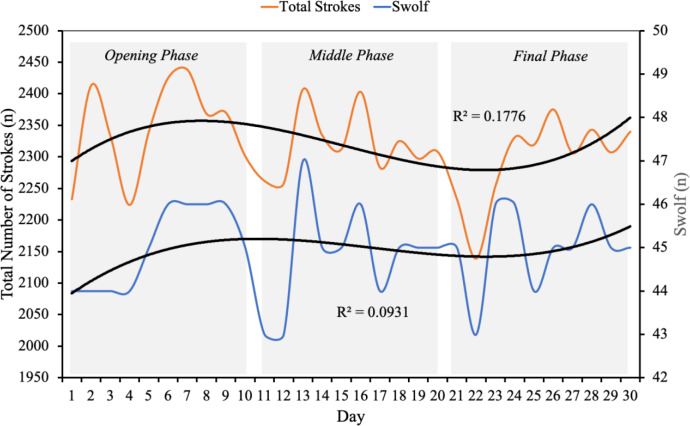
Total strokes and SWOLF index changes over the race duration.

Swimming discipline (Figure 3A(Fig. 3)) differences across race phases showed a significant reduction in high-intensity swimming time from the opening to the final phase (p = 0.042, η^2^ = 0.209). Moderate-intensity swimming time showed a near-significant trend (p = 0.052), while heart rate, pace, and energy expenditure did not significantly differ. Biomechanical parameters in swimming, including total stroke count and SWOLF index, showed increasing trends as depicted in Figure 4(Fig. 4), indicating changes in efficiency potentially related to fatigue.

It can be observed that both the total number of strokes and SWOLF increased throughout the race.

As can be seen in Figure 3B(Fig. 3), none of the observed variables showed significant differences in cycling. Energy expenditure also did not vary significantly throughout the race. However, the average pace variable showed significance (p = 0.039, η^2^ = 0.212). The results of the Tukey HSD post-hoc test showed that the average pace was significantly slower in the final phase of the race than in the initial phase (p = 0.031).

When analyzing running, significant differences were observed in running time at moderate intensity (p < 0.001, η^2^ = 0.513), high intensity (p < 0.001, η^2^ = 0.518) and average pace (p < 0.001, η^2^ = 0.603). As can be seen in Figure 3C(Fig. 3), the athlete spent significantly more time at moderate intensity (p = 0.019 and p = 0.002) and significantly less time at high intensity (p = 0.011 and p = 0.005) of running in the initial phase, compared to the middle and final phases of the race. Additionally, the athlete's average running pace in the final phase was significantly faster than in both the opening (p < 0.001) and middle (p = 0.002) phases. The middle phase pace was also significantly faster than the opening phase (p = 0.037). Although there is an increasing trend in average heart rate and energy expenditure, there were no significant differences between the different phases of the race.

**Figure 5:**
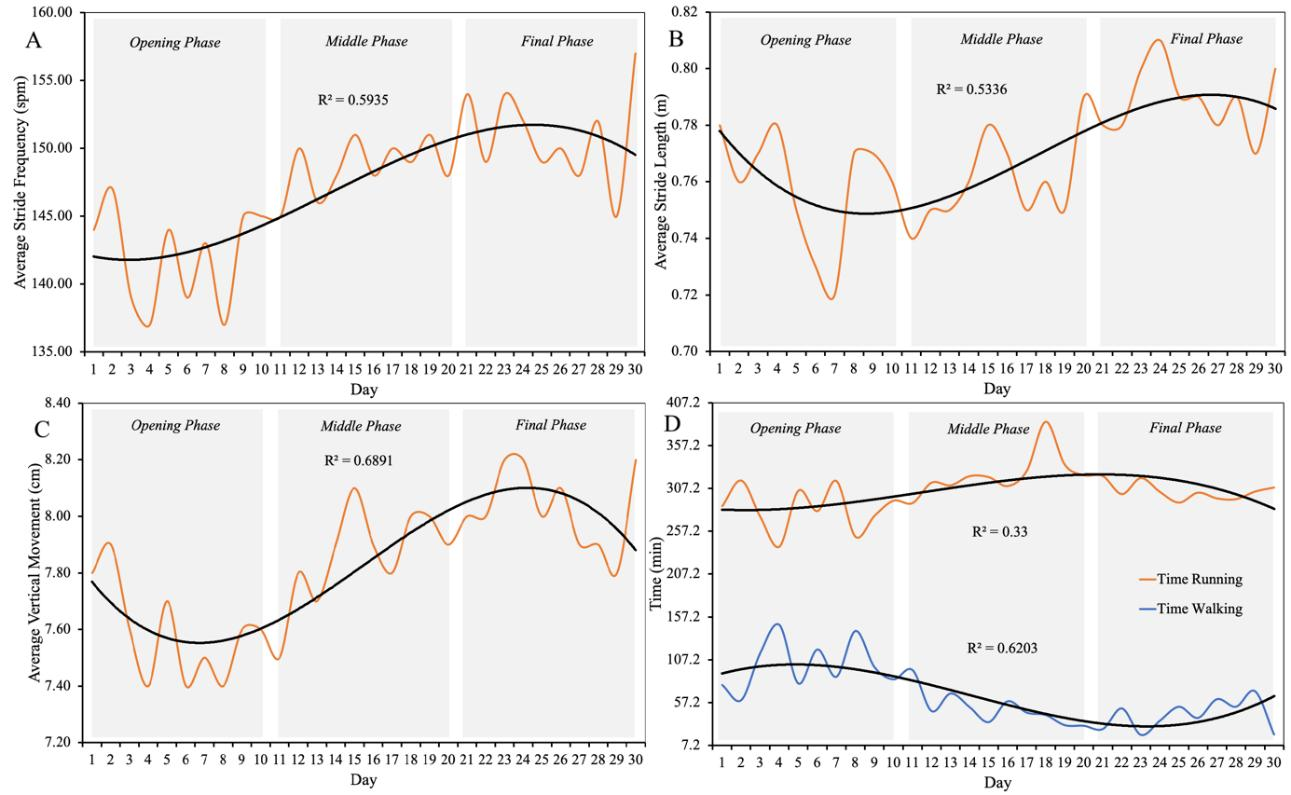
Trends in average stride frequency (*panel A*), stride length (*panel B*), vertical movement (*panel C*), time of walking and running (*panel D*) and corresponding best fit third-order polynomial regressions during the 30 days of the Triple Deca Ultra Triathlon.

As can be seen in Figure 5(Fig. 5), all three biomechanical variables decreased slightly in the opening phase of the race but then increased in the middle and final phases of the race. However, walking time decreased throughout the race, which is consistent with the overall increase in running performance.

Running biomechanics, including stride frequency, stride length, vertical movement, and time spent walking and running, are presented in Figure 5(Fig. 5).

**Figure 6:**
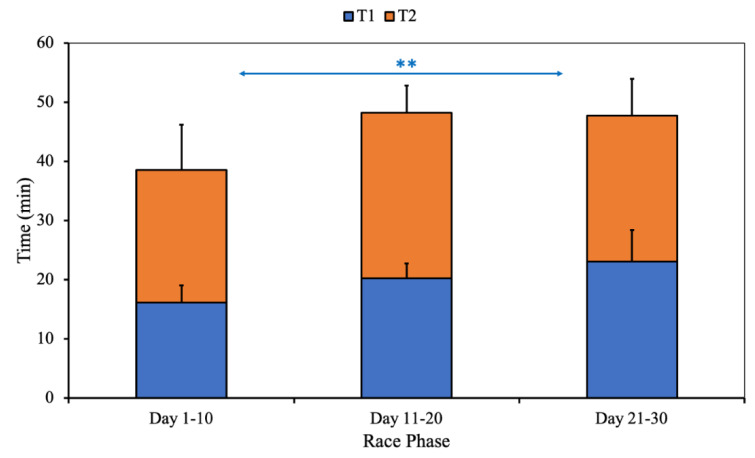
Differences in T1 and T2 between the opening phase, the middle phase and the final phase of the race.

The differences in the transition times between the different phases of the race are shown in Figure 6(Fig. 6).

As can be seen, the only significant difference was observed between the opening and final phases of the race, with T2 being significantly longer in the final phase. At T1, there were no significant differences throughout the race.

Table 1(Tab. 1) presents the correlations between average pace and physiological or race-specific variables for swimming, cycling and running across the different race phases. In the opening phase (days 1-10), significant negative correlations were found in the running between average pace and energy expenditure (r = -0.904, p < 0.01) as well as between average pace and maximum heart rate (r = -0.738, p < 0.05). Additionally, a strong positive correlation was observed between running pace and time at moderate intensity (ρ = 0.912, p < 0.01), indicating that slower running paces were associated with lower energy expenditure and heart rate, but greater time spent in moderate-intensity zones. Swimming pace was positively correlated with time at high intensity (ρ = 0.708, p < 0.05), suggesting increased stroke effort at slower speeds.

During the middle phase (days 11-20), the strongest correlations continued to be observed in running, where significant negative correlations existed between pace and energy expenditure (r = -0.642, p < 0.05), and a strong positive correlation was again found with time at moderate intensity (ρ = 0.890, p < 0.01). No significant correlations were identified in swimming or cycling during this phase.

In the final phase (days 21-30), running pace remained significantly correlated with time at moderate (ρ = 0.851, p < 0.01) and high intensity (ρ = -0.771, p < 0.01), demonstrating that as the athlete's pace increased, she spent more time running at moderate intensities and less time at high intensities. In cycling and swimming, no significant correlations were found in this phase.

For the entire race period, running pace demonstrated consistent and significant negative correlations with energy expenditure (r = -0.663, p < 0.01) and time at high intensity (ρ = -0.605, p < 0.01), and a strong positive correlation with time at moderate intensity (ρ = 0.884, p < 0.01). Swimming pace showed a weaker but significant negative correlation with maximum heart rate (r = -0.403, p < 0.05). No significant correlations were found for cycling parameters across the full race period.

Overall, the results highlight that running was the discipline most affected by physiological and pacing adaptations throughout the race, while cycling and swimming parameters demonstrated weaker or no consistent associations.

As a part of the combined approach, significant correlation analyses between variables of different split disciplines are shown in Table 2(Tab. 2) and Table 3(Tab. 3).

The correlation analysis between swimming and running variables demonstrated several significant moderate associations. Energy expenditure in swimming was positively correlated with the average running pace (r = 0.395, p = 0.031), indicating that higher energy consumption in swimming was associated with slower running speeds. Additionally, swimming energy expenditure was positively correlated with time spent at moderate intensity (r = 0.381, p = 0.038) and total running time (r = 0.383, p = 0.037), reflecting the physiological carryover of early-race exertion on later running performance.

Interestingly, time at moderate intensity in swimming was negatively associated with running average pace (r = -0.412, p = 0.024) but positively correlated with running stride frequency (r = 0.396, p = 0.030), vertical movement (r = 0.460, p = 0.011), and time running (r = 0.511, p = 0.004). These findings suggest that longer moderate-intensity swimming efforts may have encouraged more efficient or stable running biomechanics and longer running bouts. 

Conversely, time spent at high intensity in swimming was positively correlated with running pace (r = 0.374, p = 0.042) and negatively with running stride frequency (r = -0.386, p = 0.035) and vertical movement (r = -0.452, p < 0.012), implying that greater exertion in swimming was associated with compromised biomechanical efficiency in running.

Table 3(Tab. 3) highlights the correlations between cycling and running variables. Cycling energy expenditure was strongly and positively correlated with both running energy expenditure (r = 0.566, p < 0.01) and running maximum heart rate (r = 0.505, p < 0.01), indicating that higher exertion during cycling predicted higher physiological demands during running.

Moreover, cycling time at moderate intensity was negatively correlated with running energy expenditure (r = -0.450, p < 0.05) and maximum heart rate (r = -0.378, p < 0.05), while showing a positive correlation with average ground contact time in running (r = 0.390, p < 0.05). These results suggest that prolonged moderate cycling may have contributed to mechanical fatigue in running, evidenced by altered ground contact times, and possibly reduced physiological stress levels in running through better energy management.

Taken together, the combined analysis revealed moderate yet consistent cross-discipline associations, particularly highlighting how energy and pacing strategies in swimming and cycling influenced biomechanical efficiency and energy demands in running. These findings underscore the complex interplay between physiological load management in earlier disciplines and subsequent running performance in ultra-endurance triathlons.

In addition to analyzing daily relationships between swimming, cycling, and running performance, as a part of the combined approach, we conducted lagged correlation analyses to determine whether pacing and energy expenditure from the previous day could predict running performance on the following day. These analyses provide insights into pacing strategies and the carry-over effect of fatigue (Table 4(Tab. 4)).

The strongest lagged relationship was observed between the previous day's cycling pace and current running pace (r = -0.424, p = 0.022), suggesting a significant compensatory strategy: slower cycling pacing on one day is associated with faster running pacing the next day. Similarly, the previous day's running pace demonstrated a strong positive correlation with the current running pace (r = 0.644, p < 0.001), indicating that fatigue or pacing patterns in running carried over to subsequent days.

The correlations between the previous day's swimming and cycling energy expenditure and running pace, as well as total energy expenditure and total time, were not statistically significant but showed moderate trends. These findings highlight how pacing adjustments and accumulated fatigue interact across disciplines in multi-day ultra-triathlon events, particularly demonstrating strategic modulation in cycling to enable stronger running performances on the following days.

## Discussion

This case study presents unique insights into the physiological and biomechanical adaptations of the first female athlete to complete 30 consecutive IRONMAN^®^-distance triathlons in 30 days. Our findings highlight complex, discipline-specific patterns in pacing, energy expenditure, and biomechanical parameters, offering valuable information for both scientific understanding and practical coaching strategies in extreme ultra-endurance events.

### Swimming

The athlete's swimming performance remained stable across the 30 days, with minor changes in pace and energy expenditure. The slight increase in the SWOLF index and stroke count over time likely reflects fatigue-induced decreases in swimming efficiency, consistent with previous observations in prolonged ultra-endurance efforts (Puce et al., 2023[21]). However, the lack of significant changes in heart rate or energy expenditure suggests that the athlete was able to manage the swim as a controlled, steady effort without overexertion, even though the swimming efficiency (SWOLF index) slightly worsened in the Triple Deca (Maloney and Gorman, 2021[18]). This aligns with the strategy to conserve energy for subsequent disciplines (Bentley et al., 2002[2]).

### Cycling

The cycling pace showed a significant decline across the event, yet energy expenditure and heart rate remained relatively constant. Maintaining physiological characteristics, showing a relative effort similar across days, but with worsening performance, may indicate a loss of efficiency over the days, and an adjustment of the absolute intensity of cycling to maintain the physiological demand and preserve the starting conditions for the subsequent run (Phillips and Hopkins, 2020[20]). Interestingly, lagged correlation analysis suggests that slower cycling on one day was associated with improved running the next day, implying deliberate strategic modulation of cycling intensity to preserve the ability to perform well in the run (Rico Bini et al., 2022[22]).

### Running

The most remarkable finding was the progressive improvement in running pace throughout the race, accompanied by significant changes in intensity distribution. Early stages were characterized by slower running, with more time spent at moderate intensity, while the final stages showed faster paces and more time spent at higher intensities. This suggests a unique phenomenon of “delayed optimization,” where the athlete gradually adapted biomechanically and physiologically, enabling better running performance despite cumulative fatigue and a progressive increase in energy expenditure (Thompson, 2017[25]). The decrease in walking time and improvements in stride frequency and stride length in later stages further support the hypothesis of neuromuscular adaptation and increased efficiency (Hak et al., 2013[7]).

### Cross-discipline interactions

The combined analysis revealed moderate but meaningful associations between energy expenditure and biomechanical outcomes across disciplines. Higher swimming and cycling efforts appeared to influence subsequent running performance. Specifically, swimming energy expenditure correlated with slower running speeds and longer total running times, indicating cumulative fatigue effects (Lambert et al., 2004[15]). Additionally, cycling efforts impacted running ground contact time, suggesting biomechanical fatigue carryover (Rico Bini et al., 2022[22]). These findings highlight the importance of carefully balancing exertion across all disciplines, especially in multi-day events (Joyner and Cole, 2008[10]).

### Pacing and fatigue management

The athlete's ability to improve running performance, even though the slightly progressive energy expenditure and progressive fatigue may reflect a successful pacing strategy that involved controlling early intensities in swimming and cycling and progressively increasing effort in running (Boullosa et al., 2020[4]). The significant correlation between previous-day run pace and current-day performance indicates the presence of fatigue carryover, reinforcing the importance of day-to-day pacing strategies in multi-day ultra-triathlons (Kusumoto et al., 2021[14]).

### Practical implications

For ultra-endurance athletes and coaches, these findings underscore the importance of adopting flexible and responsive pacing strategies that allow for optimal distribution of energy expenditure across all disciplines. Starting conservatively supports long-term performance, particularly in multi-day events where cumulative fatigue plays a critical role. The observed improvements in running despite progressive load suggest that the body can adapt biomechanically and metabolically over time when an effort is strategically managed. Incorporating continuous monitoring of biomechanical indicators-such as stride frequency, stride length, and ground contact time-alongside physiological metrics like heart rate and energy expenditure, may provide valuable real-time feedback. These tools can aid in detecting early signs of fatigue or inefficiency, allowing for timely adjustments to training, recovery, and in-race strategies. Ultimately, a holistic and adaptive approach to pacing and load management could enhance performance and reduce the risk of breakdown in prolonged ultra-endurance challenges.

### Limitations and future research

While this case study offers valuable insights into the physiological and biomechanical adaptations of an elite female athlete during an extreme ultra-endurance event, its single-subject design limits the generalizability of the findings. Individual factors such as training background, pacing strategy, and recovery capacity may have significantly influenced the observed outcomes. To broaden applicability, future studies should include larger and more diverse samples-considering both sexes, age ranges, and levels of athletic experience-to better understand interindividual variability in adaptation patterns. Moreover, important variables such as nutrition, psychological resilience, and sleep management were not assessed in depth but likely played a critical role in sustaining performance over 30 consecutive days. Future research should explore these factors, as they may significantly affect energy availability, mental stamina, neuromuscular recovery, and day-to-day pacing decisions in multi-day ultra-endurance events.

## Conclusions

In summary, the results highlight that running was the discipline most affected by physiological and pacing adaptations throughout the race, while cycling and swimming parameters demonstrated weaker or no consistent associations. For any female athlete intending to break this record and to complete more than 30 IRONMAN^®^-distance triathlons in a row, the focus in the pre-race preparation should be to gain enough experience. During the event, the athlete should start slowly, cope with sleep deprivation and try to continuously improve performance, especially during running.

## Declaration

### Ethics approval 

The study was conducted in accordance with the recognized ethical standards according to the Declaration of Helsinki adopted in 1964 and revised in 2013. This study was approved by the Institutional Review Board of Kanton St. Gallen, Switzerland (EKSG 01/06/2010). The athlete gave her informed written consent for using all her personal data.

### Consent for publication 

Not applicable. 

### Availability of data and materials 

For this study, we have included official results and split times from the official race website. The datasets used and/or analyzed during the current study are available from the corresponding author on reasonable request.

### Competing interests 

The authors declare that they have no competing interests.

### Artificial Intelligence (AI) - Assisted Technology

The authors used AI solely for final language proofreading. All scientific content, data interpretation and conclusions were produced and verified by the authors.

### Funding 

No funding. 

### Author contributions 

Sasa Duric performed the statistical analysis and drafted the manuscript, Beat Knechtle obtained the data, Luciano Bernardes Leite, Pedro Forte, Marilia Santos Andrade, Ivan Cuk, Pantelis T. Nikolaidis, Katja Weiss, Thomas Rosemann, and Beat Knechtle helped in drafting the final version. All authors read and approved the final manuscript. 

### Acknowledgments 

We thank the athlete for providing us personal data stored in her Fenix 7 Sapphire Solar during the race.

## Figures and Tables

**Table 1:**
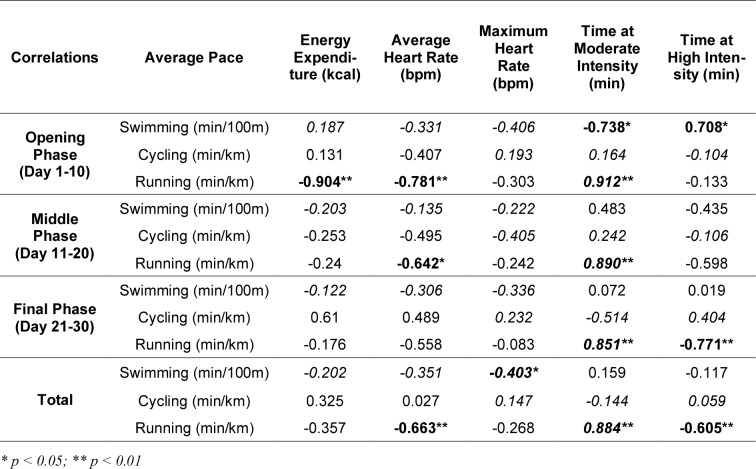
Pearson's (r) and *Spearman's (ρ)* correlation analyses between the average pace during the different disciplines and some physiological and race-specific indicators for different race phases and the whole race.

**Table 2:**
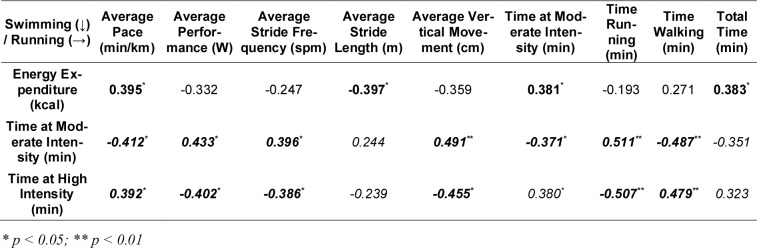
Pearson's (r) and *Spearman's (ρ)* correlation analyses between the different biomechanical, physiological and race-specific variables of swimming and running disciplines.

**Table 3:**
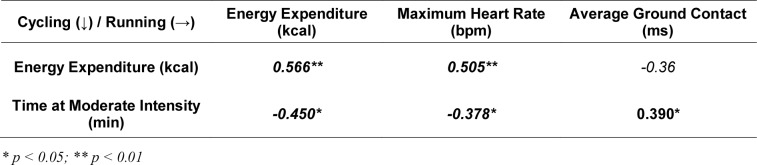
Pearson's (r) and *Spearman's (ρ)* correlation analyses between the different biomechanical, physiological and race-specific variables of cycling and running disciplines.

**Table 4:**
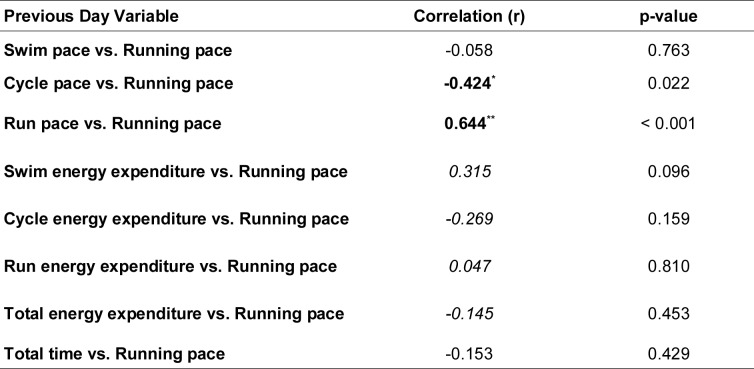
Lagged correlations between previous day variables and current running pace.
